# A Discussion of Exposure Science in the 21st Century: A Vision and a Strategy

**DOI:** 10.1289/ehp.1206170

**Published:** 2013-01-31

**Authors:** Paul J. Lioy, Kirk R. Smith

**Affiliations:** 1 Environmental and Occupational Health Sciences Institute, UMDNJ–Robert Wood Johnson Medical School and Rutgers University, Piscataway, New Jersey, USA; 2 School of Public Health, University of California, Berkeley, Berkeley, California, USA

**Keywords:** eco-exposome, exposome, exposure assessment, exposure science, National Research Council

## Abstract

Background: The National Research Council (NRC) of the National Academy of Sciences recently published the report *Exposure Science in the 21st Century: A Vision and a Strategy*. The expert committee undertaking this report included expertise from ecology, chemistry, exposure science, toxicology, public health, bioethics, engineering, medicine, and policy.

Objective: Our aim is to inform members of the scientific community in fields aligned with environmental and public health so they are more able to appreciate the full breadth of the vision and understand the framework developed in order to move the vision forward.

Discussion: Although the NRC report was commissioned by the U.S. Environmental Protection Agency and the National Institute of Environmental Health Sciences, it is solely the consensus product of the independent volunteer committee, whose findings were subject to the rigorous peer-review procedures of the NRC. In addition to reviewing the history and current status of exposure science, the report lays out a vision for the future and makes recommendations that include both short-term and long-term milestones.

Conclusion: To accomplish the vision presented in the NRC report, resources will be needed to complete studies, develop and use analyses of exposure, and build databases associated with individual and population exposures, as well as to train the next generation of exposure scientists. Important excerpts as well as paraphrased statements from the report appear in this commentary; however, the general observations and comments are our own.

The concept of exposure for nonoccupational settings other than ionizing radiation was first introduced in the early 1980s and mentioned in *Risk Assessment in the Federal Government: Managing the Process* [National Research Council (NRC) 1983], also known as the “Red Book.” It described exposure assessment as an analysis tool that was limited to evaluating single media problems. Subsequently, after a series of successes in the characterization of population exposures, a scientific field emerged—exposure science—using as its foundation field studies, laboratory experiments, and the development of fundamental equations [Lioy 1990; Ott 1995; Smith 1988a, 1988b; U.S. Environmental Protection Agency (EPA) 2009; Wallace 1987]. The first NRC committee on exposure published *Human Exposure Assessment for Airborne Pollutants: Advances and Opportunities*, also called the “White Book” (NRC 1991). That report, along with a new scientific society and federal funding for various programs, provided a path forward for the field that lasted into the 21st century. The field, now known as “Exposure Science,” continued to evolve with the founding in 1989 of the International Society for Exposure Assessment and the associated *Journal of Exposure Assessment and Environmental Epidemiology*. The society and the journal were later renamed the International Society for Exposure Science and *Journal of Exposure Science and Environmental Epidemiology;* the changes were discussed in an editorial, *“*Time for a Change: From Exposure Assessment to Exposure Science” (Lioy 2008).

Because of expanded expectations for use of exposure assessments, along with the emergence of new technologies for exposure measurements (e.g., high throughput genomic tools, personal monitors, source-to-dose modeling systems), over the past few years, it became clear that the status of the field needed to be reexamined. Guidance was needed to achieve sustainable growth in both occupational and human exposure research in order to reach the goal of improving public health. Thus, the new NRC committee (NRC Committee on Human and Environmental Exposure Science in the 21st Century) was established to provide recommendations on research activities that could assist in providing a basis for *a*) better coordination with other fields in the environmental health sciences and ecology; *b*) better approaches to address scientific, regulatory, and societal challenges; *c*) new approaches to provide exposure information for large segments of the population; and *d*) incorporation of these approaches in order to better protect humans and the ecosystem. With this in mind, the U.S. EPA and the National Institute of Environmental Health Sciences funded a new NRC study. The committee recently completed its analyses and published the “Gold Book,” *Exposure Science in the 21st Century: A Vision and a Strategy* (NRC 2012).

The overall charge to the committee was as follows:

A National Research Council committee will develop a long-range vision for exposure science and a strategy with goals and objectives for implementing the vision over the next 20 years, including a unifying conceptual framework for advancement of exposure science to study and assess human and ecologic contact with chemical, biologic, and physical stressors in their environments. In developing the vision and the strategy, the committee will consider exposure-assessment guidelines and practices used by the Environmental Protection Agency and other federal agencies, the use and development of advanced knowledge and analytic tools, and ways of incorporating more complete understanding of exposure into risk assessment, risk management, and other applications for the human health and ecologic services. The study will focus on the continuum of sources of stressors, their fate in or changes in the environment, human and ecologic exposure, and resulting doses or other relevant metrics that are relevant to outcomes of concern. (NRC 2012)

The report was envisaged to potentially be a companion to two other recent NRC reports, *Toxicity Testing in the 21st Century: A Vision and a Strategy* (NRC 2007) and *Science and Decisions: Advancing Risk Assessment* (NRC 2009). Notably, however, the charge was set much more broadly than for these other two reports, that is, to explore the entirety of exposure science including its application in ecology, not just assessment or testing.

## Exposure Science for the 21st Century

*Defining exposure science.* Exposure science is required to evaluate a wide array of problems because humans (and other species) can come into contact with physical, chemical, and biologic agents every day, as well as during natural and other catastrophic events. Consequently, the report (NRC 2012) dealt with major issues in exposure science, but it could not cover all topics. To meet its charge, the committee decided to use a focused definition of exposure science (NRC 2012):

Exposure science is defined by this committee as the collection and analysis of quantitative and qualitative information needed to understand the nature of contact between receptors (such as people or ecosystems) and physical, chemical, or biologic stressors. Exposure science strives to create a narrative that captures the spatial and temporal dimensions of exposure events with respect to acute and long-term effects on human populations and ecosystems.

This is not significantly different from the definition from Barr (2006), which has been adopted by the field of human exposure science:

Human exposure science studies human contact with chemical, physical or biological agents occurring in their environments, and advances knowledge of the mechanisms and dynamics of events either causing or preventing adverse health outcomes.

**Figure 1:**
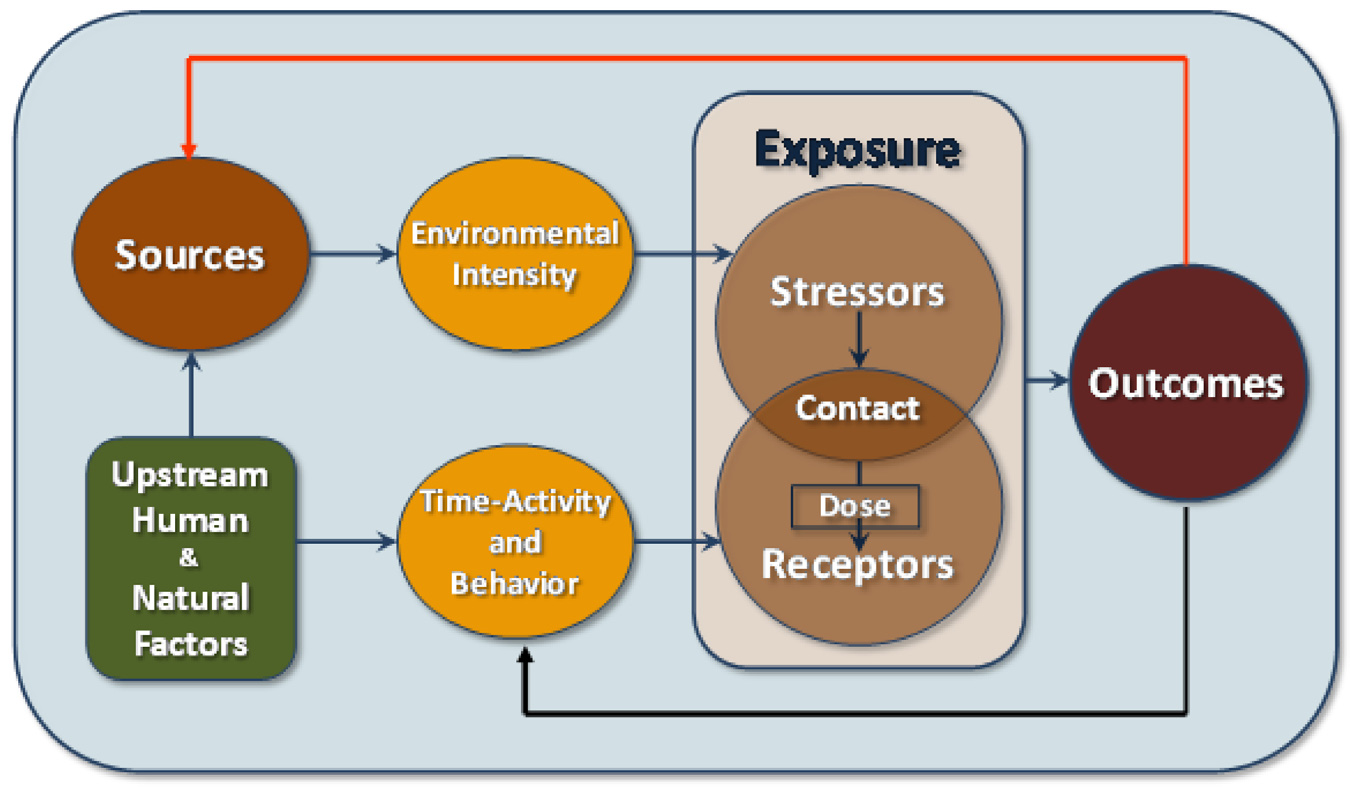
Conceptual framework showing the core elements of exposure science as related to humans and ecosystems. Reproduced from NRC (2012) with permission from the National Academy of Sciences, courtesy of the National Academies Press.

Barr’s definition emphasizes dynamics and mechanisms of contact, not just measurements. In addition, the definition can easily be amended to encompass ecological exposures. Based on the charge, the committee limited its analysis to the contact with chemical, physical, and biologic stressors and did not specifically focus on lifestyle, social conditions, and behavior, except as they affect how those stressors come into contact with people or ecosystems (Figure 1). However, the committee noted that lifestyle, social conditions, and behavior can be considered stressors in themselves and that these relationships are active areas of research. We believe that these aspects of exposure science should be considered in future evaluations of the application of exposure science to epidemiology, risk assessment, risk management, and regulations.

*From the source to the exposure to the dose*. The basic concept behind exposure science is that the point of contact between the organism at risk and the environment through which the stressor operates is the optimal point for both understanding and controlling the effect of stressors on human and ecosystem health. Exposure science links directly to the sources that might be controlled and to the internal environment of the organisms that are of concern. This point is illustrated in Figure 1. Exposure science includes the core elements of the field and provides a conceptual framework that identifies and links sources of stressors, environmental intensity, time-activity and behavior, stressors and receptors, and outcome of contact. This framework is modified from the classic source-to-dose continuum, which shows a linear relationship from source to effect (e.g., Lioy 1990; Smith 1988a). The revised framework has several important modifications:

The feedback loop shows that an outcome experienced by an individual or population could possibly be spread to others. Thus, exposure science addresses the factors that lead to both the initial exposures to stressors as well as the spread of stressors from affected populations to either another population or individuals. Some examples include diarrhea-causing organisms spread from infected humans back into the environment, leading to contact by and disease outbreaks in other humans; and sarin gas reemitted from the lungs of terrorist victims, causing exposures among emergency room health care workers.
The feedback loop includes how health outcomes among people or ecosystems can alter activities and behaviors among individuals and subpopulations that affect subsequent exposure. Thus, the health effects can change exposures, as well as vice versa. People with asthma, for example, may change their behavior and activities that affect their exposures.
The separation of time-activity into its own category emphasizes its importance in exposure science.
The explicit inclusion of “upstream” factors of exposure shows more clearly that they are integral to the field, for example, in looking at the implications of energy, land use, or transportation policies for exposures.
This framework explicitly recognizes the role of internal marker measurements as part of exposure science.

Even with the added value of each of these five modifications, the point of contact between stressors and receptors in humans and other species remains the central concept and also the goal of effective implementation of the scientific principles of exposure science. If used effectively, the updated framework shown in Figure 1 can provide opportunities for expansion of the science to deal with a broader range of critical public and environmental health issues.

A major concept discussed throughout the NRC report is internal exposure. As previously mentioned by Cohen Hubal et al. (2010), new technology can ensure that exposure science includes both external and internal markers, as appropriate. We believe this would improve links with other fields in the environmental health sciences (e.g., toxicology, epidemiology) that are applicable to risk assessment. The report notes that over the past 15 years, there has been a “greater emphasis on the use of internal markers of exposure to assist in defining exposure–response relationships.” The committee identified measurements of chemicals and metabolites in the body, oxidative modifications of DNA, and metabolomics coupled with pharmacokinetics as basic examples of internal markers of exposure. As the committee noted, the linkage of results for internal—as well as external—markers of exposure is also needed to inform the selection of relevant concentrations of stressors and chemicals for high throughput toxicity testing.

**Figure 2:**
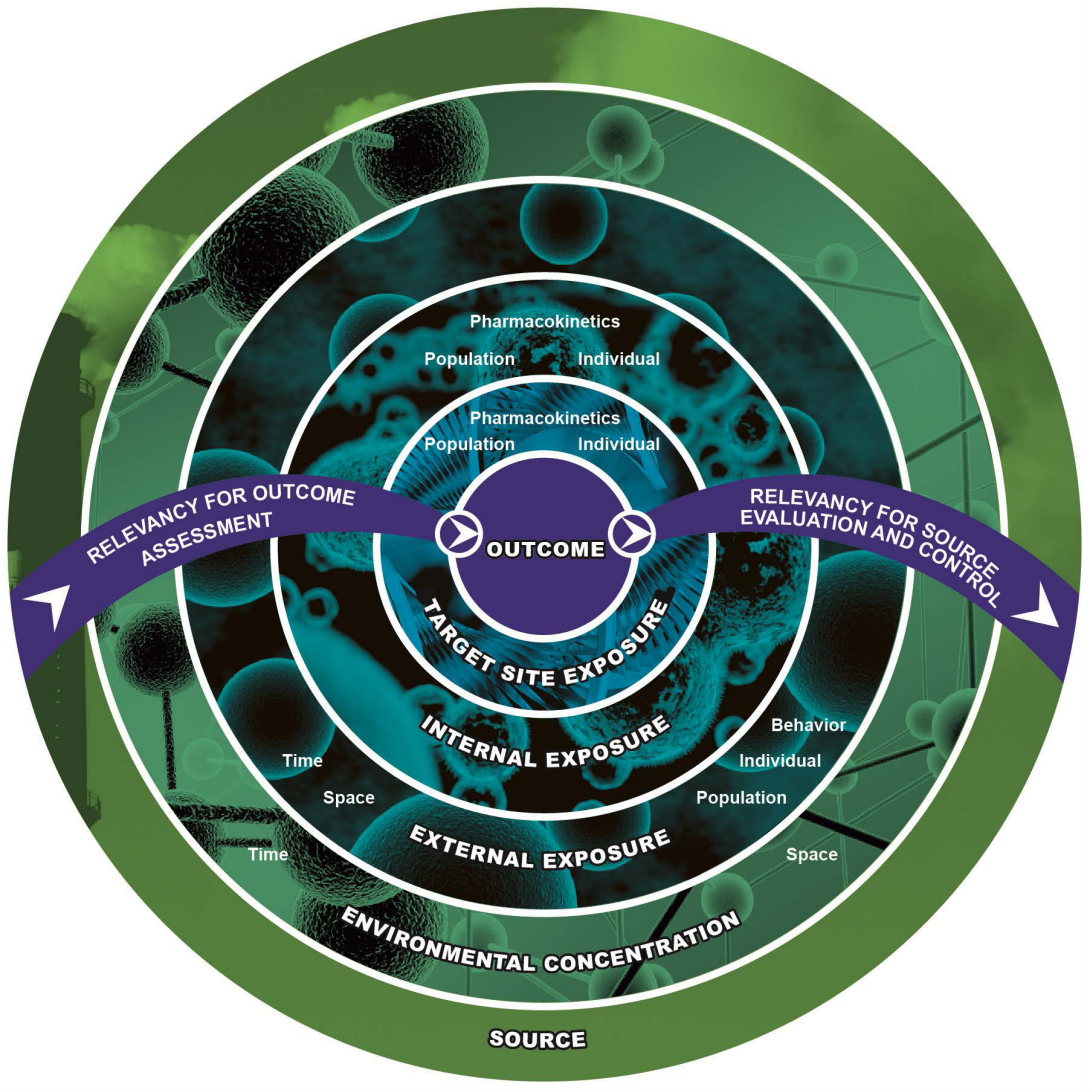
A bidirectional view of the source-to-outcome continuum for exposure science. Exposure science can be applied at any level of biologic organization. Reproduced from NRC (2012) with permission from the National Academy of Sciences, courtesy of the National Academies Press.

Figure 2 illustrates the relationship among sources, outcomes, and the dynamics of exposure. It shows internal exposure between external exposure and the dose delivered to a target site. This reflects how one person’s concern for the measurement or estimate of internal exposure can be another person’s indicator of an internal dose (Lioy 1990). More important, the expansion of exposure science to include internal markers of exposure can provide opportunities for dialog among scientists from different disciplines within the environmental health sciences. This communication will increase the probability of scientists reacting more quickly to provide treatment to a diseased population and also to use “smart” science to reduce or mitigate exposure in all parts of the world. It is also critical to note that Figure 2 acknowledges that source-to-outcome analyses are bidirectional processes. This is illustrated by the process of a toxicant exposure yielding an outcome; information on exposure outcomes can then be used to identify approaches to intervention and prevention of disease by source control or replacement, a point also noted in the report. This will lead to the development of better policies for toxicants that are already used in commerce and industrial processes.

*The eco-exposome*. The committee introduced a new concept—the “eco-exposome”—​to encapsulate the vision for advancing exposure science in the 21st century. The eco-exposome, defined as “the extension of exposure science from the point of contact between a stressor and receptor inward into the organism and outward to the general environment, including the ecosphere” (NRC 2012), thus embraces the use of both internal and external markers of exposure. This definition addresses the confusion that was beginning to evolve because the original exposome concept seemed to promote measurements that were primarily inward (i.e., internal markers of exposure) (Wild 2005, 2012). Lioy and Rappaport (2011) discussed the need to link internal and external markers of human exposure as part of the exposome; this concept was also discussed by Wild (2012). The eco-exposome concept also captures the important link outward from the organism (i.e., human or other species) to single or multipollutant sources, which is critical in making effective control decisions. As noted throughout the report (NRC 2012), the eco-exposome narrative will improve the collection of “exposure information for making informed decisions on human and ecosystem health protection.” We are, however, anticipating a significant dialogue on this issue because, as with the exposome, it is a proposed concept.

The report (NRC 2012) discusses many new measurement and detection tools and mathematical modeling systems, and notes that the eco-exposome opens new paths to encourage the continued development of innovative tools to address the spectrum of scientific questions facing many segments of public and environmental health.

*Achieving the exposure science vision*. The committee (NRC 2012) identified four major activities and research points required to achieve its vision of the extension of exposure science from the point of contact between stressor and receptor inward into the organism and outward to the general environment, including the ecosphere:

*Assess and mitigate exposures quickly in the face of emerging environmental-health threats and natural and human-caused disasters*. For example, this requires expanding techniques for rapid measurement of single and multiple stressors on diverse geographic, temporal, and biologic scales. That includes developing more portable instruments and new techniques in biologic and environmental monitoring to enable faster identification of chemical, biologic, and physical stressors affecting humans or ecosystems.

*Predict and anticipate human and ecologic exposures related to existing and emerging threats*. Development of models or modeling systems will enable us to anticipate and characterize exposures that had not been previously considered. For example, predictive tools will enable development of exposure information on thousands of chemicals that are now in widespread use and enable informed safety assessments of existing and new applications for them. In addition, strategic use of such diverse information as structural properties of chemicals, non-targeted environmental surveillance, biomonitoring, and modeling tools, are needed for identification and quantification of relevant exposures that may pose a threat to ecosystems or human health.

*Customize solutions that are scaled to identify problems*. As stated in *Science and Decisions: Advancing Risk Assessment* (NRC 2009), the first step in a risk assessment should involve defining the scope of the assessment in the context of the decision that needs to be made. Adaptive exposure assessments could facilitate that approach by tailoring the level of detail to the problem that needs to be addressed. Such an assessment may take various forms, including very narrowly focused studies, assessments that evaluate exposures to multiple stressors to facilitate cumulative risk assessment, or assessments that focus on vulnerable or susceptible populations.

*Engage stakeholders associated with the development, review, and use of exposure-science information, including regulatory and health agencies and groups that might be disproportionately affected by exposures*–that is, engage broader audiences in ways that contribute to problem formulation, monitoring and data collection, access to data, and development of decision-making tools. Ultimately, the scientific results derived from the research will empower individuals, communities, and agencies to prevent and reduce exposures and to address environmental disparities.

These key aspects of the vision provide a firm foundation for filling the boxes and flow of information associated with Figure 1, and indicate that, during the completion of the research and other scientific activities, it is necessary to provide various stakeholders access to data in order to eventually mitigate or prevent future exposures. Finally, it brings in to focus the need to be prepared to quickly evaluate and mitigate population and occupational multipollutant or single-pollutant exposures during disasters, including terrorist attacks or military actions.

Although the report does not describe available measurement methods, it supports moving away from dependence on default “exposure factors” in the preparation of risk and environmental impact assessments and increasing expectations for actual measurement of important variables, and then using these measurements to model exposure for specific situations. The report continues to support the concept of the “exposure pyramid” used in epidemiologic and risk assessment studies, in which even simple metrics, such as the percentage of households exposed, can sometimes be a useful cost-effective exposure measure. At the same time, investigators should consider the trade-offs in going to more costly and intrusive measurement platforms, such as personal monitoring and biomonitoring, in terms of reduction of exposure misclassification and improving intervention and prevention strategies (NRC 1991).

To achieve the vision, programs that include smart science approaches should be designed and developed to to provide information and analyses that can be applied to solve current and unanticipated problems (e.g., natural events and terrorist attacks). Further, data acquisition must be coupled with significant opportunities to assemble and interpret data (Lioy 2010).

Informatics is an important tool for assembling data from the results obtained from the collection of internal (biomonitoring) and external (personal and microenvironmental monitoring) from, for example, high throughput instrumental analyses and continuous or smartphone-based technology, respectively. However, the ability to use and interpret such data is still in its infancy. To address this issue the committee (NRC 2012) strongly acknowledged that implementation of its vision will “depend on development and cultivation of scientists, engineers, and technical experts with experience in multiple fields to educate the next generation of exposure scientists.” In the United States, this will require “an increase in the number of academic predoctoral and postdoctoral training programs” and provide “short-term training and certification programs” to meet immediate needs. In the era of reduced funding, the report indicates that agencies must develop transagency coordination and resources for all aspects of exposure science research and education. Clearly, such programs can lay a foundation for the development of similar activities worldwide, both in developed and underdeveloped countries.

**Figure 3:**
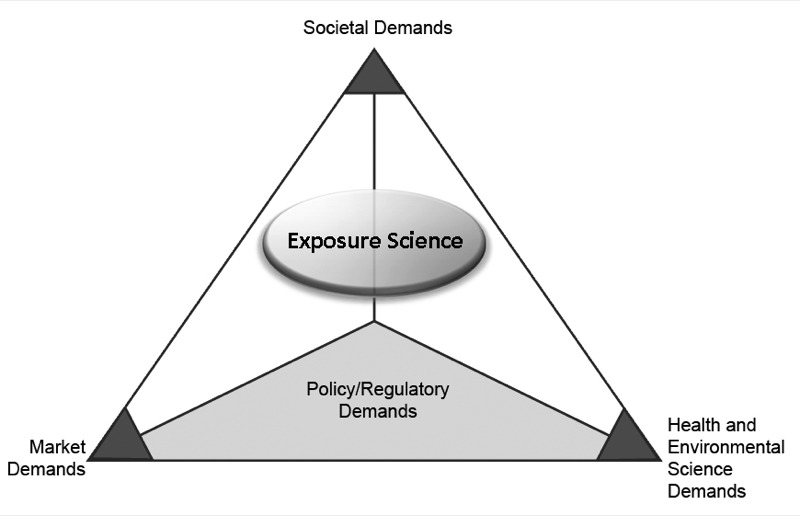
The four major demands for exposure science. Reproduced from NRC (2012) with permission from the National Academy of Sciences, courtesy of the National Academies Press.

The report (NRC 2012) provides examples of demands for research that can be conducted by exposure science, which are summarized in Figure 3. Of the four types of demands (societal, market, health and environmental, and policy/regulation), the most well-known are the health and environmental demands, but the others are critical in a national perspective and—during the realization of the vision—an international perspective. Market demands will include control or replacement of materials in consumer products, before sale as well as after sale and use. Societal demands will include aspirations from individuals and communities to understand and participate in the reduction of single or multiroute exposures and consequential environmental health risks. However, because the demands transcend geopolitical boundaries, they can be drivers for addressing both domestic and global problems [e.g., energy and fuels (Smith 1988b, 1993; Smith et al. 2012)].

**Figure 4:**
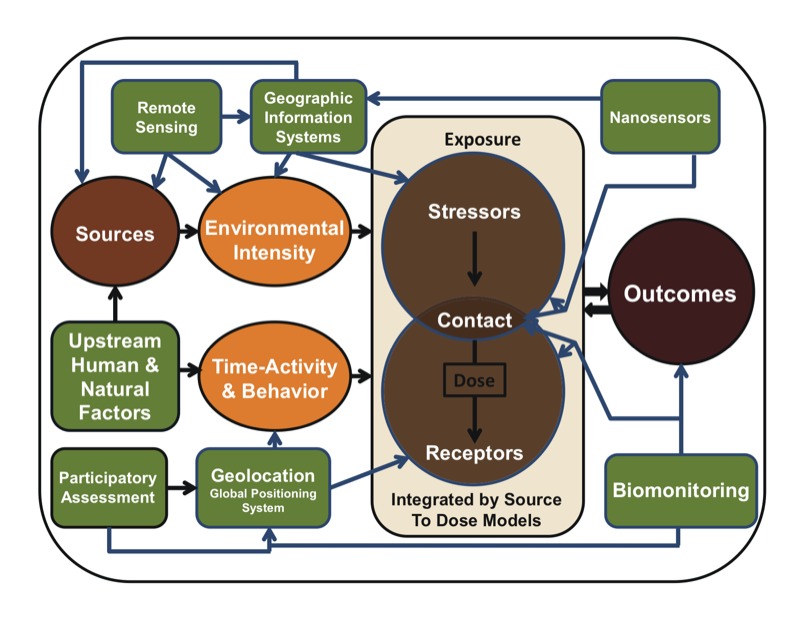
Selected scientific and technological advances for measuring and monitoring considered in relation to the conceptual framework presented in Figure 1. Reproduced from NRC (2012) with permission from the National Academy of Sciences, courtesy of the National Academies Press.

The overall scope of the NRC report’s recommendations for expanding the research activities and types of data required for the field are summarized in Figure 4, which illustrates how some of the new tools described in detail in the report (e.g., Chapter 5) can be placed within the framework to improve the collection of new exposure data and development of models to improve linkages to outcome assessment. The types of tools mentioned in Figure 4 are examples that were evaluated for inclusion in the report; depending on the route of exposure and the agents of concern, the needs for tools associated with the boxes may change (NRC 2012). As transagency opportunities are discussed in the United States and considered by other agencies around the world, augmented versions of the graphic would be useful to identify critical needs and paths in exposure science for environmental and ecological health, emergency response, risk assessment and risk management.

## Conclusion

Although broadly framed to include ecological and human impacts, the NRC report’s charge was to focus on issues of particular concern to the United States. Thus, it does not directly address, for example, the many special exposure issues of developing countries, where environmental health threats are the greatest at all levels—from household to community to global (Lim et al. 2012; Smith and Ezzati 2005). This could well be the grist of a future report.

In addition, the report (NRC 2012) does not address the past and potential use of regulation and management of exposures from either single or multiple routes of contact in both environmental and occupational settings. More sophisticated use of exposure science represents potential opportunities for protecting more workers and members of the public at less expense than current practices, through smart regulation and management that also maintains the highest standards of equity. This would be an excellent subject for a future report.

With the mandate to focus on the future, the report also does not explore the still considerable improvements in current and past epidemiologic and risk studies that are promised by more complete application of some of the classic concepts of exposure science, such as “total exposure,” which would capture all routes, places, times, and durations of exposure. An example is the growing evidence of apparently nonthreshold—and sometimes even supralinear—effects at what were once considered low exposure levels: the lower the level of detected exposure, the higher the potential for exposure misclassification due to multiple pathways and routes of contact. This also would be a timely subject for assessment.

Finally, the vision presented in the NRC report to mitigate and/or prevent future impacts of chemical, physical, and biologic stressors is both bold and achievable. However, resources are required to complete the investigations necessary for developing and using external and internal analyses of exposure and for building databases associated with exposures to individuals and large populations. Only then can source-to-effect modeling systems simulate the dynamics and mechanisms of contact with chemical, physical, and biologic stressors. The results can be used to mitigate exposures to stressors associated with single or multiple routes of contact. Concurrently, the next generation of exposure scientists needs to be trained to implement the vision and embrace and quantitatively elaborate on the concept of the eco-exposome. Such an approach can be used to examine and solve human and environmental health problems around the world.
